# Characteristics of dry eye associated with autoimmune diseases

**DOI:** 10.1007/s10384-025-01172-9

**Published:** 2025-03-17

**Authors:** Ayano Yoshimura, Yuka Hosotani, Nahomi Masuda, Fumi Gomi

**Affiliations:** Department of Ophthalmology, Hyogo Medical University, 1-1, Mukogawa-cho, Nishinomiya, Hyogo 663-8501 Japan

**Keywords:** Sjogren’s syndrome, Dry eye, Autoimmune disease, Fluorescein breakup patterns, Anti-SS-A antibody

## Abstract

**Purpose:**

To investigate the characteristics of dry eye in patients with autoimmune disease.

**Study Design:**

Retrospective clinical study.

**Methods:**

Two hundred and fifty-two eyes from 252 patients (12 men and 240 women, mean age 59.8 ±15.2) with autoimmune disease and dry eye were enrolled. Patients were divided into three groups: primary Sjogren’s syndrome, secondary Sjogren’s syndrome, and autoimmune disease without Sjogren’s syndrome.

**Results:**

Among all cases, the mean fluorescein breakup time (FBUT) was 2.7 ± 1.6 s, the van Bijesterveld corneal score was 0.8 ± 0.8, the conjunctival score was 1.5 ± 1.7; and the Schirmer 1 test value was 8.4 ± 8.2 mm. Among all cases, the fluorescein breakup pattern (FBUPs) was tear-deficiency-type dry eye in 43% of eyes (area 11%, line 32%), and short FBUT-type dry eye in 57% (dimple 31%, spot 10%, random 16%). Conjunctival scores showed significant positive correlations with anti-SS-A and anti-SS-B antibody titers in the primary and secondary Sjogren’s syndrome groups.

**Conclusion:**

More than half of the dry eyes associated with autoimmune diseases were short FBUT-type. Patients with autoimmune disease should be aware of dry eye, even without a diagnosis of Sjogren’s syndrome.

## Introduction

Autoimmune disease occurs when the body’s immune system loses its tolerance to its own tissue antigens, which results in pathological immune responses that can lead to tissue and organ damage. Systemic autoimmune diseases include systemic lupus erythematosus (SLE), rheumatoid arthritis (RA) [1], systemic sclerosis [2], Sjogren’s syndrome (SS) [3], mixed connective tissue disease [4], and primary biliary cholangitis [5]. Many studies point out that patients with autoimmune disease not only have systemic effects, but also ocular manifestations. Dry eye is a common manifestation in autoimmune disease [6–8], and SS is known to be a typical autoimmune disease causing aqueous-deficiency-type dry eye, with its diagnostic criteria including a Schirmer test value of 5 mm or less and keratoconjunctival epithelial disorder [3].

A recent report by the Asia Dry Eye Society proposed new definitions and diagnostic criteria for dry eye [9], with short breakup time (BUT) dry eye [10–12] being characterized by abnormal BUT and severe symptoms, despite a normal volume of tear fluid and minimal ocular surface epithelial damage.

Along with these proposed definitions, new concepts for the diagnosis and therapy of dry eye, called tear film-oriented diagnosis (TFOD) and tear film therapy (TFOT), have been advanced in Japan [9, 13, 14]. Applying TFOD, Yokoi et al. classified fluorescein breakup patterns (FBUPs) into five types [14, 15]. This classification is useful for diagnosing the insufficient component of the ocular surface that is responsible for tear film breakup, classifying dry eye subtypes, and selecting the best available topical therapy for dry eye.

Although dry eye research from the perspective of TFOD and TFOT is progressing in Japan, there are only few reports on the use of TFOD in the classification of dry eye associated with autoimmune diseases. Here we report on the characteristics of dry eye associated with autoimmune diseases.

## Materials and methods

### Subjects

This study describes a retrospective analysis of dry eye in patients with autoimmune disease (SLE, RA, systemic sclerosis, SS, mixed connective tissue disease, primary biliary cholangitis, and autoimmune hepatitis) who presented at Hyogo Medical University Hospital ophthalmology department between April 2013 and June 2021. The respective autoimmune disease was diagnosed by a physician and all patients were enrolled in the National Health Insurance system. Either the eye with the lower value in the Schirmer 1 test, or the right eye whenever the Schirmer 1 test gave equivalent values, was chosen for examination. The research protocol followed the guidelines of the Declaration of Helsinki and was approved by the Institutional Review Board of Hyogo Medical University (Review Board Number 2377). Informed patient consent was obtained in the form of an opt-out on the website. The exclusion criteria included ocular surface disorders such as active keratitis or conjunctivitis, neurological abnormalities, contact lens use, insertion of a lacrimal punctum plug, and eye or lid surgery within 6 months prior to the study. After applying the exclusion criteria, 252 patients with dry eye (12 men and 240 women, mean age 59.8 ±15.2, age range 18–92 years) were enrolled as eligible subjects. After thorough examinations, those eyes that showed qualitative and/or quantitative tear abnormalities were further analyzed to elucidate the characteristics of tear abnormalities associated with autoimmune disease.

### Classification according to the presence or absence of Sjogren’s syndrome

Patients were classified into the following three groups: (1) primary SS, (2) secondary SS, and (3) non-SS, autoimmune disease without SS.

### Assessment of objective signs of tear abnormalities

For each patient, a series of less-to-more invasive ophthalmic examinations was performed, including measurements of fluorescein breakup time (FBUT), classification of FBUP scoring of corneal and conjunctival fluorescein staining, and the Schirmer 1 test (ST1). For the evaluation of fluorescein staining and assessment of FBUT and FBUP, a slit-lamp biomicroscope with a cobalt blue filter was used; we note that yellow filters (blue-free filters) are not used in our hospital. After two drops of saline solution were placed onto a fluorescein test strip (Ayumi Pharmaceutical Corporation), the strip was shaken vigorously. In order not to increase the patient’s tear volume inadvertently, strict attention was paid to ensure that the strip gently touched the center of the lid margin to stain the tear film with fluorescein. This procedure was followed by several natural blinks. FBUT was then measured as the time (in seconds) until the first appearance of a dark spot in the fluorescein-stained precorneal tear film while the eye was kept open. FBUT was measured three times with a stopwatch and the average was calculated. In these three FBUT measurements, FBUPs were examined three times according to a previously reported classification, and were classified into one of five types: area, spot, line, dimple, and random break [14, 15].

FBUP and FBUT evaluations were followed by assessment of fluorescein staining of the ocular surface epithelium. For the assessment of conjunctival epithelial fluorescein staining, the bulbar conjunctiva was divided into nasal and temporal portions. The van Bijesterveld scoring system [16] was used to score the corneal and conjunctival epithelial damage in each portion with a value from 0 to 3, and the total score was calculated. Accordingly, the overall corneal damage level was scored on a scale of 0–3, and the conjunctival epithelial damage level was scored from 0 to 6 points. In this study, the ST1 was performed by means of standard Schirmer test strips without topical anesthesia. The strips were placed for 5 minutes in the temporal third of the lower conjunctival fornix of each patient, and were then removed. The length (in mm) of the filter paper that had been wetted was recorded.

### Measurement of autoantibodies

Anti-SS-A antibody, anti-SS-B antibody, antinuclear antibody (ANA), and rheumatoid factor (RF) were measured by intravenous blood sampling. When the anti-SS-A and anti-SS-B antibodies were measured by the enzyme-linked immunosorbent assay method, they were converted to the equivalent of the chemiluminescent immunoassay method measurement using the conversion formula published by the SRL company: anti-SS-A: Y = 2.174 X − 32.221; anti-SS-B: Y = 2.750 X − 4.841. Values below the measurement limit were recorded as 0, and values above the limit were recorded as the upper limit maximum value. An ANA score of 40 or more was taken to indicate a positive test, and the rate of positive tests within each group was calculated.

### Diagnosis and classification of dry eye

According to Japanese diagnostic criteria, those with some subjective symptoms and an FBUT equal to or less than 5 seconds are diagnosed with dry eye [17]. Subjective symptoms include ocular discomfort and abnormal visual function, assessed by a detailed interview. The current Asia Dry Eye Society report proposes a simple classification of dry eye based on the concept of TFOD, and suggests that there are three types of dry eye: aqueous-deficient, decreased wettability, and increased evaporation. It is suggested that these three types respectively coincide with the problems in different layers: aqueous, membrane-associated mucins, and lipid/secretory mucin [9, 13, 14, 18]. In the present study, based on the concept of TFOD, area and line breaks are classified as the aqueous-deficient type, and spot, dimple, and random breaks are classified as short FBUT-type, with this classification encompassing both decreased wettability and increased evaporation types. If line break and spot break are seen at the same time, the classification of line break is given priority [19].

### Outcome measures

The outcome measures consisted of the following ophthalmic and serological objective assessments: (1) measurement of FBUT [20] and classification of FBUPs [14, 15], (2) van Bijesterveld scores [16], (3) ST1 without topical anesthesia, and (4) measurements of anti-SS-A antibody, anti-SS-B antibody, ANA, and RF. Ten minutes were left between assessments (1), (2), and (3).

### Statistical analysis

Statistical analyses were performed using Excel add in Statcel4 software (Microsoft and OMS). Continuous variables are presented as mean and SD for normally distributed data and as median and interquartile range for non-normally distributed data. All results are expressed as mean ± standard deviation. The Mann–Whitney U test was used to assess differences in FBUT, the van Bijesterveld score, ST1, and autoantibodies. Spearman’s rank correlation was used to estimate the correlations between each autoantibody’s titer and the objective scores. All p values were two-tailed, and a p value of < 0.05 was accepted as a significant difference.

## Results

The primary SS group accounted for 40% (100/252) of the patients (mean age 61.9 ± 14.5), the secondary SS group for 40% (102/252; mean age 58.5 ± 15.2), and the non-SS group for 20% (50/252; mean age 58.4 ± 16.2; Table 1).

**Table 1:** Patient demographics

Demographic	Primary SS (n=100)	Secondary SS (n=102)	Non-SS (n=50)
Women, n (%)	96 (96%)	99 (97%)	45 (90%)
Mean age ± SD	61.9±14.5	58.5±15.2	58.4±16.2
Other autoimmune disease, n (%)			
RA	–	73 (71.6%)	31 (62%)
SLE	–	20 (19.6%)	12 (24%)
SSc	–	14 (13.7%)	6 (12%)
PBC	–	4 (3.9%)	2 (4%)
AIH	–	13 (12.7%)	4 (8%)
MCTD	–	3 (2.9%)	1 (2%)
Other	–	6 (5.9%)	2 (4%)

Patients may have more than one disease. *AIH* autoimmune hepatitis, *MCTD* mixed connective tissue disease, *PBC* primary biliary cholangitis, *SS* Sjogren’s syndrome, *RA* rheumatoid arthritis, *SLE* systemic lupus erythematosus, *SSc* systemic sclerosis

RA accounted for more than half of all autoimmune diseases other than SS in both the secondary SS and non-SS groups (secondary SS: 71.6%, non-SS: 62%), followed by SLE (secondary SS: 19.6%, non-SS: 24%; Table 1). It should also be noted that some patients had multiple autoimmune diseases.

**Fig. 1:**
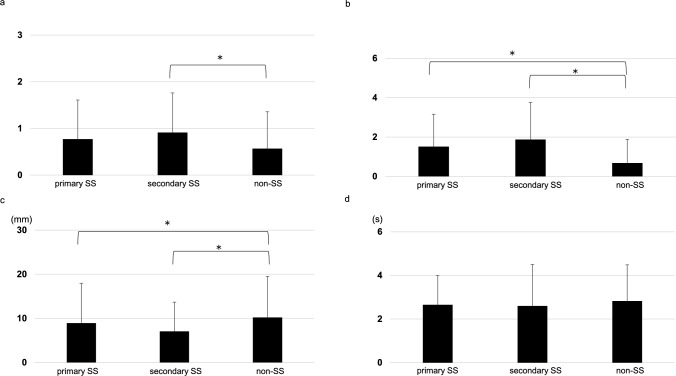
Comparison of objective findings between the three patient groups. **a** corneal score, **b** conjunctival score, **c** Schirmer 1 test, d. breakup time. **a** There was a significant difference in corneal score between secondary SS and non-SS. **b** There were significant differences in conjunctival score between primary SS, secondary SS, and non-SS groups. **c** There were significant differences in ST1 between primary SS, secondary SS, and non-SS. d. There were no significant differences between the three groups. Data are expressed as mean ± SD, *p < 0.05 (Mann–Whitney U test). SS, Sjogren’s syndrome

The mean corneal scores in the primary SS, secondary SS, and non-SS groups were 0.77 ± 0.84, 0.92 ± 0.85, and 0.56 ± 0.76, respectively. There was a significant difference in corneal score between the secondary SS and non-SS groups (*p* = 0.041; Fig. 1a). The mean conjunctival scores in the primary SS, secondary SS, and non-SS groups were 1.52 ± 1.65, 1.88 ± 1.90, and 0.69 ± 1.19, respectively. There were significant differences in conjunctival scores between the primary SS and non-SS groups, and between the secondary SS and non-SS groups (*p* = 0.003, < 0.001, respectively; Fig. 1b). The mean ST1 values of the primary SS, secondary SS, and non-SS groups were 8.9 ± 9.0, 7.0 ± 6.7, and 10.0 ± 8.7 mm, respectively. There were significant differences in ST1 between the primary SS and non-SS groups, and between the secondary SS and non-SS groups (*p* = 0.01, 0.004, respectively; Fig. 1c). The FBUT scores of the primary SS, secondary SS, and non-SS groups were 2.7 ± 1.3, 2.5 ± 1.7, and 3.0 ± 2.0 seconds, respectively, with no significant differences between the three groups (Fig. 1d).

**Fig. 2:**
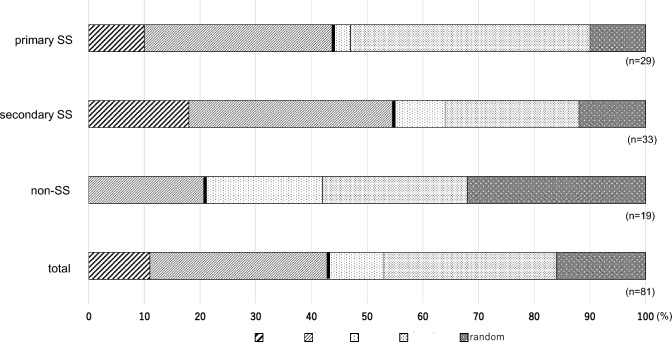
Classification of fluorescein breakup patterns in patients with autoimmune disease. No area break was observed in the non-SS group. Area break and line break, which reflect tear-deficiency-type dry eye, represented more than half of breaks in the secondary SS group, but less than half in total. More than half of the patients’ breakup patterns were classified as short breakup-time-type dry eye. *SS* Sjogren’s syndrome

Observation of FBUPs was performed in 81 cases. In the primary SS group, the FBUPs included 12 cases of dimple break (41%, 12/29), 10 of line break (34%, 10/29), 3 of area break (10%, 3/29), 3 of random break (10%, 3/29), and 1 of spot break (3%, 1/29). In the secondary SS group, the FBUPs included 12 cases of line break (36%, 12/33), 8 of dimple break (24%, 8/33), 6 of area break (18%, 6/33), 4 of random break (12%, 4/33), and 3 of spot break (9%, 3/33). In the non-SS group, the FBUPs included 6 cases of random break (32%, 6/19), 5 of dimple break (26%, 5/19), 4 of line break (21%, 4/19), and 4 of spot break (21%, 4/19). No patient showed an area break (Fig. 2). Overall, area and line breaks in the aqueous-deficient type showed frequencies of 11% (9/81) and 32% (26/81), respectively, dimple and spot breaks representing the decreased wettability type showed frequencies of 31% (25/81) and 10% (8/81), respectively, and random breaks indicating the increased evaporation type showed a frequency of 16% (13/81) (Fig. 2).

**Fig. 3:**
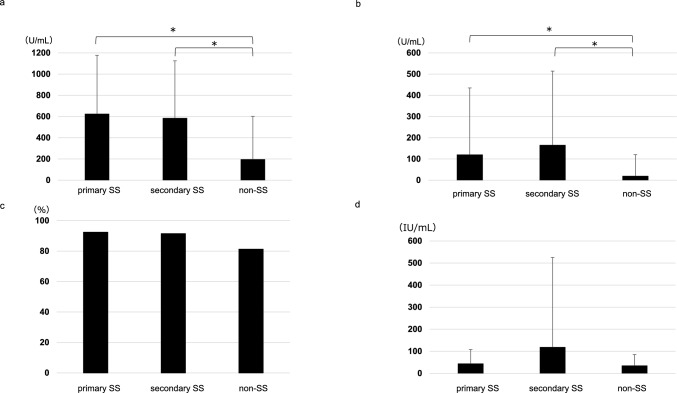
Comparisons of various autoantibodies between the three patient groups. **a** anti-SS-A antibody, **b** anti-SS-B antibody, **c** positive rate of antinuclear antibody, d. rheumatoid factor. Anti-SS-A and anti-SS-B antibodies showed significant differences between primary SS, secondary SS, and non-SS. The positive rates of antinuclear antibody and rheumatoid factor showed no significant differences between the three groups. Data are expressed as mean ± SD, *p < 0.05 (Mann–Whitney U test). *SS* Sjogren’s syndrome

The mean anti-SS-A antibody values of the primary SS, secondary SS, and non-SS groups were 626.5 ± 547.7, 586.3 ± 536.6, and 197.6 ± 399.1 U/mL, respectively. There were significant differences between the primary SS and non-SS groups, and between the secondary SS and non-SS groups (*p* = 0.024, < 0.001, respectively; Fig. 3a). The mean anti-SS-B antibody values of the primary SS, secondary SS, and non-SS groups were 121.0 ± 311.7, 165.9 ± 346.2, and 20.1 ± 98.6 U/mL, respectively. There were significant differences between the primary SS and non-SS groups, and between the secondary SS and non-SS groups (p = 0.006, 0.001, respectively; Fig. 3b). The ANA-positive rates of the primary SS, secondary SS, and non-SS groups were 92.4%, 91.5%, and 81.3%, respectively. There were no significant differences between the three groups (Fig. 3c). The RFs of the primary SS, secondary SS, and non-SS groups were 44.6 ± 63.2, 119.5 ± 403.5, and 35.6 ± 49.1 IU/mL, respectively. There were no significant differences between the three groups (Fig. 3d).

There was no significant correlation between ST1 and any of the autoimmune antibodies, nor between ST1 and the conjunctival scores (primary SS: *r* = 0.183, *p* = 0.077; secondary SS: *r* = 0.21, *p* = 0.051; non-SS group: *r* = 0.09, *p* = 0.635). In the primary SS group, there were significant positive correlations between anti-SS-A antibody and corneal scores (*r* = 0.281, *p* = 0.005), and anti-SS-A and anti-SS-B antibodies titers and conjunctival scores (SS-A: *r* = 0.391, *p* < 0.001; SS-B: *r* = 0.250, *p* = 0.013). In the secondary SS group, there were significant positive correlations between all antibodies and conjunctival scores (SS-A: *r* = 0.352, *p* < 0.001; SS-B: *r* = 0.314, *p* = 0.002; RF: *r* = 0.280, *p* = 0.005). In the non-SS group, conjunctival scores showed significant positive correlations with anti-SS-A antibody and RF (SS-A: *r* = 0.293, *p* = 0.040; RF: *r* = 0.271, *p* = 0.029) (Table 2).

**Table 2:** Correlations between objective ophthalmological findings and autoantibodies

	Primary SS		Secondary SS		Non-SS	
	*r*	*p* value*	*r*	*p* value*	*r*	*p* value*
ST1						
Anti-SS-A Ab	– 0.103	0.306	0.071	0.474	– 0.225	0.170
Anti-SS-B Ab	– 0.040	0.723	– 0.092	0.353	– 0.080	0.686
RF	0.133	0.188	0.083	0.201	0.022	0.893
Corneal score						
Anti-SS-A Ab	0.281	**0.005**	0.008	0.932	– 0.010	0.509
Anti-SS-B Ab	0.146	0.145	0.118	0.235	– 0.003	0.986
RF	0.156	0.121	0.189	0.058	– 0.179	0.235
Conjunctival score						
Anti-SS-A Ab	0.391	**<0.001**	0.352	**<0.001**	0.293	**0.040**
Anti-SS-B Ab	0.250	**0.013**	0.314	**0.002**	0.101	0.495
RF	0.136	0.177	0.280	**0.005**	0.271	**0.029**

Bold values indicate statistical significance (*p* < 0.05). ^*^Spearman’s rank correlation test. *RF* rheumatoid factor, *SS* Sjogren’s syndrome, *ST*1 Schirmer 1 test. Significant are in value [bold]

## Discussion

Dry eye is a common manifestation in autoimmune diseases [6–8]. Fujita et al. report that 10% of RA patients met the Japanese criteria for SS, and even in a non-SS group, 90% of patients were diagnosed with probable dry eye [21]. In patients with SLE, the prevalence of dry eye was 16%, with 12% of the patients also meeting the criteria for SS [22]. Although there are many reports on the relationships between autoimmune diseases and dry eye, the diagnostic criteria for dry eye differ from report to report, and in many reports the diagnosis of dry eye was based solely on the patient’s subjective symptoms. There are few reports discussing the FBUPs or subtype classifications of dry eye associated with autoimmune diseases.

In this study, we classified patients into three groups according to the presence or absence of SS. SS is a typical disease causing aqueous-deficient-type dry eye. It is an autoimmune disorder characterized by immune-mediated destruction of the salivary and lacrimal glands, with subsequent development of decreased tear production [3, 4, 23, 24]. Thus, considering the currently published reports, we predicted that aqueous-deficient type dry eye would represent the majority of the SS groups, but to the contrary, half of the SS groups were classified as short FBUT-type dry eye.

Guannan et al. report that in autoimmune disease patients, conjunctival epithelial cells undergo apoptosis, resulting in a decrease in the number of goblet cells, even without dry eye [8]. Compared with a non-autoimmune disease dry eye group, the patients in a dry eye autoimmune disease group showed significantly higher corneal staining counts and ocular surface inflammation [25]. In an SS group, the concentrations of tear cytokines such as IL-17, TNF-a, and IL-6 were found to be significantly higher than in a non-SS dry eye group and control subjects [26]. Furthermore, the bulbar conjunctival lissamine green staining score was found to be significantly greater in SS dry eye patients than in non-SS dry eye patients, and greater conjunctival staining was associated with a reduction in tear MUC5AC [27].

In this study, the Schirmer test score was relatively high, with a result of 6 mm or more in the Sjögren’s syndrome group. A study on patients with RA found that the duration of RA was positively correlated with Schirmer test and staining scores [28]. In patients with autoimmune disease, even if lacrimal gland function is not impaired, secretory mucin disorders may occur because of a reduction in secreted-type mucin caused by a decrease in goblet cells, and destruction of membrane-type mucin and galectin-3 due to inflammation. As a result, we speculated that in such patients, tear film stability would be decreased and short FBUT-type dry eye would occur. In addition, although patients underwent ophthalmological examination to confirm their diagnosis, the duration of the disease was considered to be short, and therefore dysfunction of the lacrimal gland may be less advanced than in patients with longer-term disease. In patients with autoimmune disease, it is necessary to suspect the presence of short FBUT-type dry eye, even without the presence of tear volume loss. If dry eye can be diagnosed by measuring FBUT and observing FBUPs, it should be possible to use this information to contribute to improving the quality of life of patients with autoimmune disease.

In primary SS, the presence of anti-SS-A antibody was found to be significantly correlated with Rose Bengal scores [29]. Our results are similar to these findings: in the primary SS group, there were significant positive correlations between anti-SS-A antibody and corneal scores, and anti-SS-A and anti-SS-B antibody titers and conjunctival scores. In patients with SS, the higher the autoantibody titer the stronger the conjunctival epithelial disorder, although we found no correlation between the autoantibody titer and tear secretion ability. In the present study, no correlation was found between Schirmer test values and anti-SS-A and anti-SS-B antibodies or conjunctival score. In dry eye, increased friction due to blinking is thought to affect conjunctival scores [30, 31]. Although it was difficult to assess friction in this study because blinking was not taken into account, it is not likely that conjunctival damage is more common because of low tear fluid volume.

The limitations of this study include that it is a retrospective study, and cases with no results for the tear fluid test or cases in which autoimmune disease was diagnosed in internal medicine but the ophthalmology was not examined were excluded. Therefore, there is a possibility that the subject recruitment is biased. In addition, because we searched for patients using the National Health Insurance system by disease name, there may have been cases where the true diagnosis was different. Although the objective signs of tear abnormalities used the data at the time of the first visit to our hospital, some cases had already been treated with eye drops at other clinics, and the objective signs of tear results may have been modified. Observation of FBUP was only performed in 81 cases in this study. In this regard, the first ophthalmologist differed on different days of the week and they did not necessarily have to examine FBUP at the time of consultation, which may have contributed to the low number of cases. In addition, because the results of the findings are from multiple physicians, the method of taking the findings may not have been consistent, and further prospective detailed studies are needed. Patients with autoimmune diseases can develop dry eye with or without a diagnosis of SS, and short FBUT-type dry eye is common and should be diagnosed and treated according to TFOD and TFOT.
